# Transcriptome Analysis of Neisseria gonorrhoeae during Natural Infection Reveals Differential Expression of Antibiotic Resistance Determinants between Men and Women

**DOI:** 10.1128/mSphereDirect.00312-18

**Published:** 2018-06-27

**Authors:** Kathleen Nudel, Ryan McClure, Matthew Moreau, Emma Briars, A. Jeanine Abrams, Brian Tjaden, Xiao-Hong Su, David Trees, Peter A. Rice, Paola Massari, Caroline A. Genco

**Affiliations:** a Department of Immunology, Tufts University School of Medicine, Boston, Massachusetts, USA; b Biological Sciences Division, Pacific Northwest National Laboratory, Richland, Washington, USA; c Program in Bioinformatics, Boston University, Boston, Massachusetts, USA; d Centers for Disease Control and Prevention, Atlanta, Georgia, USA; e Department of Computer Science, Wellesley College, Wellesley, Massachusetts, USA; f Nanjing STD Clinic, Institute of Dermatology, Chinese Academy of Medical Sciences and Peking Union Medical College, Nanjing, China; g Department of Medicine, Division of Infectious Diseases and Immunology, University of Massachusetts Medical School, Worcester, Massachusetts, USA; University of Kentucky; University of Virginia; University of Wisconsin—Madison

**Keywords:** Neisseria gonorrhoeae, RNA-seq, antibiotic resistance, human mucosal infection

## Abstract

Neisseria gonorrhoeae is a bacterial pathogen responsible for the sexually transmitted infection gonorrhea. Emergence of antimicrobial resistance (AMR) of N. gonorrhoeae worldwide has resulted in limited therapeutic choices for this infection. Men who seek treatment often have symptomatic urethritis; in contrast, gonococcal cervicitis in women is usually minimally symptomatic, but may progress to pelvic inflammatory disease. Previously, we reported the first analysis of gonococcal transcriptome expression determined in secretions from women with cervical infection. Here, we defined gonococcal global transcriptional responses in urethral specimens from men with symptomatic urethritis and compared these with transcriptional responses in specimens obtained from women with cervical infections and *in vitro*-grown N. gonorrhoeae isolates. This is the first comprehensive comparison of gonococcal gene expression in infected men and women. RNA sequencing analysis revealed that 9.4% of gonococcal genes showed increased expression exclusively in men and included genes involved in host immune cell interactions, while 4.3% showed increased expression exclusively in women and included phage-associated genes. Infected men and women displayed comparable antibiotic-resistant genotypes and *in vitro* phenotypes, but a 4-fold higher expression of the Mtr efflux pump-related genes was observed in men. These results suggest that expression of AMR genes is programed genotypically and also driven by sex-specific environments. Collectively, our results indicate that distinct N. gonorrhoeae gene expression signatures are detected during genital infection in men and women. We propose that therapeutic strategies could target sex-specific differences in expression of antibiotic resistance genes.

**IMPORTANCE** Recent emergence of antimicrobial resistance of Neisseria gonorrhoeae worldwide has resulted in limited therapeutic choices for treatment of infections caused by this organism. We performed global transcriptomic analysis of N. gonorrhoeae in subjects with gonorrhea who attended a Nanjing, China, sexually transmitted infection (STI) clinic, where antimicrobial resistance of N. gonorrhoeae is high and increasing. We found that N. gonorrhoeae transcriptional responses to infection differed in genital specimens taken from men and women, particularly antibiotic resistance gene expression, which was increased in men. These sex-specific findings may provide a new approach to guide therapeutic interventions and preventive measures that are also sex specific while providing additional insight to address antimicrobial resistance of N. gonorrhoeae.

## Abstract (precis)

Recent emergence of antimicrobial resistance of Neisseria gonorrhoeae worldwide has resulted in limited therapeutic choices for treatment of infections caused by this organism. We performed global transcriptomic analysis of N. gonorrhoeae in subjects with gonorrhea who attended a Nanjing, China, sexually transmitted infection (STI) clinic, where antimicrobial resistance of N. gonorrhoeae is high and increasing. We found that N. gonorrhoeae transcriptional responses to infection differed in genital specimens taken from men and women, particularly antibiotic resistance gene expression, which was increased in men. These sex-specific findings may provide a new approach to guide therapeutic interventions and preventive measures that are also sex specific while providing additional insight to address antimicrobial resistance of N. gonorrhoeae.

## INTRODUCTION

Neisseria gonorrhoeae is responsible for the sexually transmitted infection (STI) gonorrhea. Rates of gonococcal infection in the United States have increased 46% since 2011, and approximately 450,000 cases of gonorrhea were reported to the U.S. Centers for Disease Control and Prevention in 2015. N. gonorrhoeae is the second most common reportable bacterial STI in the United States (1). Infection with N. gonorrhoeae also contributes significantly to global STI morbidity and is responsible for 78 million cases each year (2). For the past 8 decades, gonorrhea has been treated successfully with antibiotics, despite patterns of resistance, but over the last several years, multidrug-resistant and untreatable strains have begun to emerge worldwide. Resistant strains include isolates from both Eastern and Western Europe (3, 4), Asia (5–7), and recently the United States (8–10). This rise in antibiotic-resistant strains may be linked, in part, to an increase in availability of over-the-counter antibiotics, particularly in many parts of Asia. New strategies for combating this disease are necessary, as evidenced by increased efforts to develop gonococcal vaccines (11, 12) and new drug regimens (7, 13, 14).

In humans, the only natural host for N. gonorrhoeae, symptomatic responses to infection are unique to men and women. While most infected men remain asymptomatic (15), those who develop symptoms often show robust inflammation characterized by purulent urethral discharge accompanied by large numbers of polymorphonuclear leukocytes (PMNs). In women, gonococcal infections are asymptomatic 50 to 80% of the time (16) or are accompanied by nonspecific symptoms such as vaginal discharge (17). The presence of cervical mucus or microscopic PMN counts of ≥10 seen microscopically on an oil immersion field does not correlate well with gonococcal infection in the absence of coinfecting agents (18). Manifestations of gonococcal infection may be driven by environmental factors specifically associated with the genital tracts of men and women: these may include biofilm formation (19, 20); the influence of the microbiome, particularly in women (21–25); and both direct and indirect molecular interactions between gonococci and specific host cells from men or women (26–30).

Gonococcal pathogenesis and host immune responses have been studied principally *in vitro* and in animal models (31), both of which may not faithfully replicate differences in infection between men and women. Historically, the only human model of gonococcal infection is based on genital experimental challenge of male volunteers, which has provided valuable information on gonococcal virulence factors, but is limited by the duration of the experimental infection and is not applicable to women (32, 33). A better understanding of gonococcal pathogenesis during natural mucosal infection, which is less well studied, is critical to implement biological strategies to treat and prevent infection. Previous work from our group using a gene-specific microarray and reverse transcription-quantitative PCR (qRT-PCR) analysis demonstrated that at least 20 gonococcal iron-regulated genes were expressed during human mucosal infection in men and women (34, 35). Recently, we also examined the complete N. gonorrhoeae transcriptome using cervicovaginal lavage specimens of infected women attending an STI clinic in China where antibiotic-resistant N. gonorrhoeae is prevalent (36, 37). We reported that a large portion of the gonococcal genome is expressed (65% of protein-coding genes) and regulated during infection (305 genes regulated compared to *in vitro* growth) (38). In the present study, we examined gonococcal transcriptomes from men attending the same clinic and compared bacterial gene expression and regulation in infected men with expression and regulation in infected women. The resulting data revealed sex-specific adaptation of genes involved in host-pathogen interactions and phage and antibiotic resistance.

## RESULTS

### Description of subjects.

The male cohort comprised six male subjects (average age, 34 years) attending the Nanjing (China) Sexually Transmitted Disease (STD) Clinic, who presented with urethral discharge and/or dysuria and were diagnosed with N. gonorrhoeae infection (Table 1). Four men were coinfected with Chlamydia trachomatis and/or Mycoplasma genitalium, three had a history of prior gonococcal infection, and three had self-administered antibiotics prior to their clinic visit (Table 1). In contrast, none of the seven enrolled women had a history of gonococcal infection or had taken antibiotics prior to their clinic visit (see Table S1 in the supplemental material). Two gonococcal strains were isolated from a man and a woman who comprised a single dyad (subjects M-4 and F-6, respectively).

10.1128/mSphereDirect.00312-18.3 TABLE S1  Characteristics of female subjects. Download TABLE S1, DOCX file, 0.02 MB. Copyright © 2018 Nudel et al. 2018 Nudel et al. This content is distributed under the terms of the Creative Commons Attribution 4.0 International license.

**TABLE 1:** Characteristics of male subjects with gonococcal urethritis

Subject	Age (yr)	No. of days with symptoms	Other STI microbe(s)	Urethral PMN score^a^	Prior gonococcal infection	ABX in last 30 days^b^
M-1	27	4	C. trachomatis	+++	Yes	Yes
M-2	37	21	None	+++	No	No
M-3	46	3	None	+++	Yes	No
M-4	28	3	M. genitalium	+++	No	Yes
M-5	34	15	M. genitalium, C. trachomatis	+++	Yes	Yes
M-6	32	1	C. trachomatis	++	No	No

a Number of PMNs per oil immersion field indicated: ++, 5 to 9; +++, ≥10.

b Self-administered antibiotics (ABX) prior to diagnosis in the STD clinic.

### Gonococcal gene expression in the human genital tract.

RNA was extracted from male urethral or cervicovaginal lavage specimens, and individual cDNA reads were aligned to N. gonorrhoeae strain FA1090 using Rockhopper (39). Total gene expression was reported in reads per kilobase per million (RPKM). On average, 8.1% of the total RNA from male urethral specimens aligned to the N. gonorrhoeae FA1090 genome and 42.5% to the human genome; 4.3% of total RNA from cervicovaginal lavages aligned to FA1090 and 45% to the human genome.

**FIG 1:**
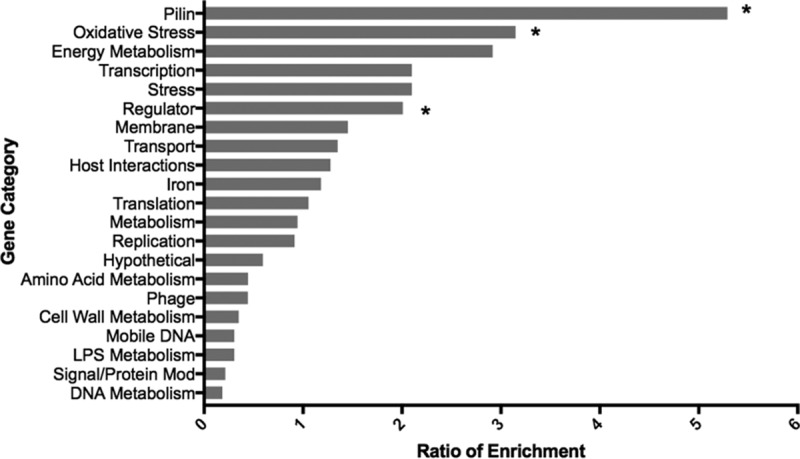
Categorization of the top 200 N. gonorrhoeae genes expressed during natural infection in men. Expression of gonococcal genes in 6 infected men was averaged, and the top 200 genes were categorized based on characteristics indicated for N. gonorrhoeae strain FA1090 available in NCBI. Categories are shown on the *y* axis, and the ratio of enrichment (% of genes of a given functional category in RNA-seq data set/% of genes assigned to that functional category in whole gonococcal genome) is shown on the *x* axis. For clarity, ribosomal protein, tRNA, and rRNA genes were removed. Asterisks indicate that enrichment was significant (*P* ≤ 0.05) by Fisher’s exact test.

Expression of 1,725 gonococcal genes (approximately 65% of the gonococcal genome) was detected in male urethral specimens. High expression of a core set of genes that encoded oxidative stress products (e.g., peroxiredoxin/glutaredoxin), housekeeping genes (e.g., the gene coding for elongation factor Tu), outer membrane protein genes (e.g., genes coding for Rmp [Omp3] and porin P.IB), and hypothetical protein genes were identified across male specimens (see Table S3 in Data Set S1 in the supplemental material). A more detailed analysis of the top 200 gonococcal genes (approximately 10% of total genome) that were expressed in all six male urethral secretions (in aggregate) was carried out by determining the ratio of gene enrichment and segregation into broad categories. Despite some variability in RPKM values among subjects, significantly enriched categories included genes coding for pilin and oxidative stress and regulatory proteins (Fig. 1), which reflected a common pattern of high gene expression during natural infection.

10.1128/mSphereDirect.00312-18.5 DATA SET S1 Supplemental Tables S3 to S7. Table S3 shows the top 10 most highly expressed genes in each male subject. Table S4 contains the composite RPKM values for each gene in men *in vivo* versus *in vitro*. Table S5 contains the composite RPKM values for significantly different noncoding RNA genes in men *in vivo* versus *in vitro*. Table S6 contains the composite RPKM values for significantly different predictive sRNAs in men *in vivo* versus *in vitro* and men *in vivo* versus women *in vivo*. Table S7 contains the composite RPKM values for each gene in men versus women. Download DATA SET S1, XLSX file, 0.2 MB. Copyright © 2018 Nudel et al. 2018 Nudel et al. This content is distributed under the terms of the Creative Commons Attribution 4.0 International license.

### Gonococcal gene regulation in the male genital tract.

**FIG 2:**
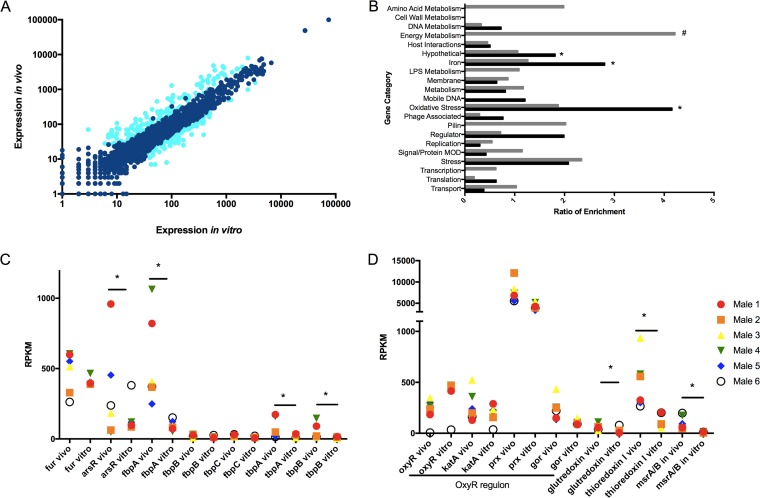
Comparison of N. gonorrhoeae gene expression in infected men *in vivo* and isolates grown *in vitro*. (A) Composite gene expression levels in urethral specimens (*n =* 6) on the *y* axis plotted against composite expression of corresponding gonococcal isolates (*n =* 6) grown *in vitro* (*x* axis). Data are shown as log_10_ expression (RPKM) levels. The genes indicated in light blue had *q* values of ≤0.05 and ≥2-fold changes *in vivo* (versus *in vitro*). (B) Functional enrichment of genes with statistically significant changes in expression under *in vivo* and *in vitro* conditions. Genes were differentially expressed if the *q* value was ≤0.05 and the fold change was ≥2. Black bars represent functional enrichment of genes with increased expression *in vivo*. Gray bars represent functional enrichment of genes with decreased expression *in vivo*. Categories are shown on the *y* axis, and the ratio of enrichment (% of genes of a given functional category in RNA-seq data set/% of genes assigned to that functional category in whole gonococcal genome) is shown on the *x* axis. Ribosomal protein, tRNA, and rRNA genes were removed for clarity. *, significant enrichment among genes increased *in vivo* (versus *in vitro*), and #, significant enrichment among genes decreased *in vivo*, with *P* ≤ 0.05, by Fisher’s exact test. (C and D) Expression levels of iron genes (C) and oxidative stress genes (D) under *in vivo* and *in vitro* conditions. (Six male specimens [*in vivo*] and the corresponding 6 isolates [*in vitro*] are shown in the same color in adjacent columns.) *, *q* ≤ 0.05.

Our earlier studies had demonstrated that approximately 17% of gonococcal genes were regulated *in vivo* during cervical infection in women (38). Gonococcal gene expression in the male genital tract was compared to expression of matched infecting strains grown *in vitro* by analysis of transcriptome sequencing (RNA-seq) data sets for individual strains and for the composite data sets. Genes that displayed a significant difference in expression under *in vivo* versus *in vitro* conditions were selected for further analysis based on the following criteria: a ≥2-fold difference in RPKM value, a *q* (false-discovery rate) value of ≤0.05, and expression greater than 10 RPKM under at least one condition (see Table S4 in Data Set S1). The majority of genes were expressed similarly *in vivo* and *in vitro*, but approximately 4.2% of the total number of genes detected (79 genes) revealed increased expression *in vivo* compared to that observed *in vitro*; expression of 5.2% (99 genes) was decreased *in vivo* compared to that observed *in vitro* (Fig. 2A).

Regulation of gonococcal gene expression was then examined by functional enrichment analysis (Fig. 2B). Genes involved in iron and oxidative stress pathways and genes encoding hypothetical proteins were among those significantly enriched and were more highly expressed *in vivo* than *in vitro*. For example, expression of certain iron-scavenging genes (e.g., *fbpA*, *tbpA*, and *tbpB*) and of the *fur*-controlled regulator *arsR* was significantly higher *in vivo* than *in vitro*, although variance in *arsR in vivo* expression was large (Fig. 2C). This observation suggests that the male genital tract is iron depleted (similar to the female genital tract [38]). Genes involved in energy metabolism were also significantly enriched but had decreased expression *in vivo* (Fig. 2B). Among the downregulated energy metabolism genes was the *nuoA–N* operon (NGO1737–1751) (40), involved in aerobic respiration; its overall decreased expression *in vivo* may suggest a mechanism for bacterial survival in an anaerobic environment (41).

Among the enriched oxidative stress genes increased *in vivo*, we observed a significant increase in a glutaredoxin family protein gene (NGO0031), the thioredoxin I (*trx1*) gene (NGO0652), and *msrAB* (NGO2059) (Fig. 2D). The family of glutaredoxin proteins function as glutathione-dependent reductases (42); *msrAB* plays a role in reducing oxidized methionines to reactive peptides (43) and *trx1* has been shown to respond to oxidation and is under control of the Fur regulon (44, 45). While expression of the well-studied OxyR regulon (*oxyR*, *prx*, *kat*, and* gor*) was not significantly different in *in vivo* samples compared to cultures grown *in vitro*, we observed decreased expression of the OxyR repressor *in vivo*, with increased expression of genes within the OxyR regulon (*prx*, *kat*, and* gor*). This notable trend further suggests that the gonococcus is exposed to reactive oxygen species during mucosal infection in men (Fig. 2D) (46, 47).

Several gonococcal noncoding RNAs were also regulated during infection in men. These included 4 rRNAs and 29 tRNAs, which were expressed at higher levels during infection compared to growth *in vitro*, except for NGO t31, an arginine tRNA that was expressed more highly during *in vitro* growth (see Table S5 in Data Set S1). Putative small RNAs (sRNAs: defined as expression representing an intergenic region of the complementary strand of a known open reading frame [ORF] and 30 to 250 nucleotides in length) were also regulated during infection; 29 sRNAs were increased *in vivo* compared to *in vitro*, and 21 sRNAs were decreased *in vivo* (see Table S6 in Data Set S1). Among these sRNAs, several have been described previously (48, 49).

### Comparison of gonococcal gene signatures in the male and female genital tracts.

**FIG 3:**
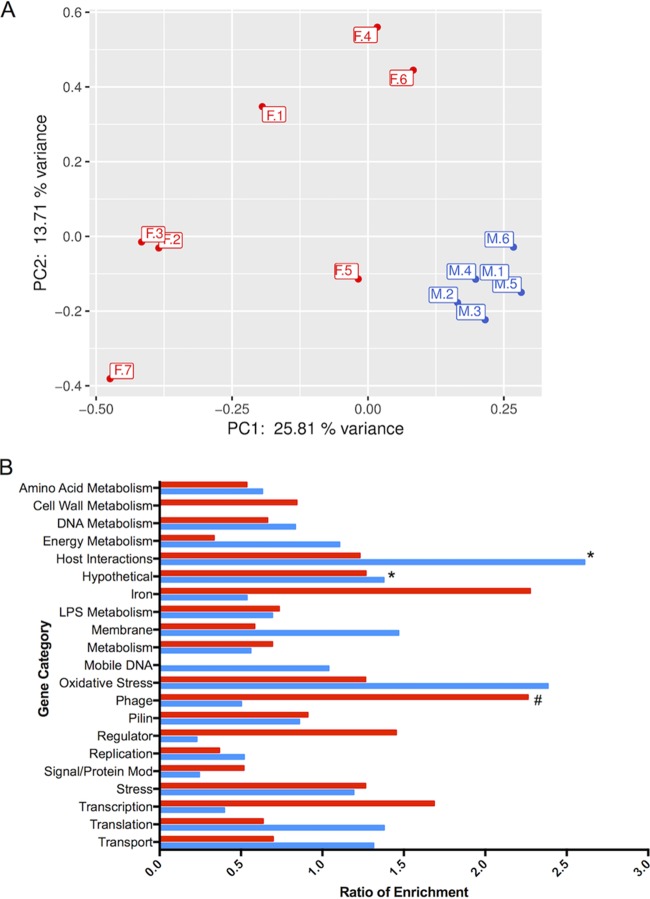
Comparison of N. gonorrhoeae gene expression during infection in men and women *in vivo*. (A) Variance in global expression in each specimen from men and women. The first principal component (PC1 [*x* axis]) had a variance of ~26%, and the second principal component (PC2 [*y* axis]) had a variance of ~14%. (B) Functional enrichment of genes with significant changes in expression levels in men and women. Genes were differentially expressed if the *q* value was ≤0.05 and the fold change was ≥2. Blue bars represent genes with increased expression *in vivo* in men compared to women. Red bars represent genes with increased expression *in vivo* in women compared to men. *, significant enrichment among genes increased *in vivo* in men; #, significant enrichment among genes increased *in vivo* in women.

Building on gonococcal transcriptome data in infected men and women, we examined whether site-specific, environmental conditions influenced expression of gonococcal genes during infection. A comparative transcriptome analysis was carried out using the 6 urethral and 7 cervicovaginal specimens. We performed principal-component analysis (PCA) to assess whether global *in vivo* gene expression revealed transcriptional signatures specific for infections in men or women (Fig. 3A). PCA showed a clear distinction between gonococcal genes expressed in specimens from men and women, with high variance among the 7 specimens from women compared to low variance in men, as evidenced by tight clustering when the first two principal components were plotted against each other (Fig. 3A). Gene expression analysis of the composite data sets was also performed; parameters set for defining differential gene regulation were a ≥2-fold change in RPKM, a *q* value of ≤0.05, and expression greater than 10 RPKM under at least one condition (see Table S5 in Data Set S1). Of 1,879 genes expressed, 176 (9.4%) were increased in men compared to women and 80 (4.3%) were decreased in men, indicating that nearly 14% of gonococcal genes were differentially regulated during gonococcal infection in the male and female genital tracts (see Table S7 in Data Set S1).

A functional enrichment analysis of gene categories performed on male and female specimens revealed that phage-associated genes were significantly enriched in female specimens (Fig. 3B). While female specimens had higher expression of a broad range of phage types (e.g., double-stranded and filamentous single-stranded phages), male specimens had higher expression in the subset of double-stranded DNA prophages (see Fig. S1A in the supplemental material) (50). Other highly expressed categories enriched in male specimens included genes that encoded hypothetical proteins and proteins involved in host interactions (Fig. 3B). Iron and oxidative stress genes that were differentially expressed in male and female specimens included the ferredoxin gene (NGO1859), a TonB receptor protein gene (NGO1205), and *fetA* (NGO2093). (*fetA* is regulated by MpeR and induced under low-iron conditions [51].) These genes all manifested increased expression in women (Fig. S1B). In contrast, expression of the oxidative stress gene *bfrB* was increased in male specimens; other oxidative stress genes were similarly expressed in both men and women (Fig. S1C), suggesting that exposure to oxidative stress occurs in both the male and female genital tracts.

10.1128/mSphereDirect.00312-18.1 FIG S1  Phage expression, iron regulation, and oxidative response genes in the male and female genital tracts. (A) Expression levels of phage genes are shown on the *x* axis and represent male urethral samples (blue) and female cervicovaginal lavage samples (red). (B and C) Expression levels of significantly differentially expressed iron genes (B) and oxidative stress genes (C). *, gene more highly expressed in the male genital tract; #, gene more highly expressed in the female genital tract (*q* value of <0.05). Red and blue X’s indicate the male and female counterparts of the dyad. Download FIG S1, TIF file, 0.8 MB. Copyright © 2018 Nudel et al. 2018 Nudel et al. This content is distributed under the terms of the Creative Commons Attribution 4.0 International license.

Regulation of several gonococcal sRNAs was also observed. A significant change in expression of 119 sRNAs was observed in specimens obtained from men and women: 28 were expressed at higher levels in male specimens and 91 in female specimens. Previously identified sRNAs showing regulation based on sex included smRNA4, smRNA5, and smRNA8 (49), *fnrS* (a small RNA induced under anaerobic conditions [44]), and the iron-repressed sRNA encoding NrrF (52) (see Table S6 in Data Set S1). In addition, 45 tRNAs showed differential regulation, with all being more highly expressed in female patients than in male patients (see Table S5 in Data Set S1).

### Expression of antimicrobial resistance genes.

Gonococcal antimicrobial resistance (AMR) is a growing concern and is common in strains from the eastern regions of the world, including China. We examined phenotypic and genotypic AMR patterns from our cohort and found that all the isolates exhibited resistance to penicillin, tetracycline, and ciprofloxacin, with one isolate showing resistance to azithromycin (Table 2). One isolate also showed reduced susceptibility to ceftriaxone. Strains isolated from women exhibited similar antimicrobial susceptibility patterns (Table 2). These results were similar to AMR patterns of male urethritis strains reported earlier (2011 to 2012) from the Nanjing STD Clinic (53).

**TABLE 2:**
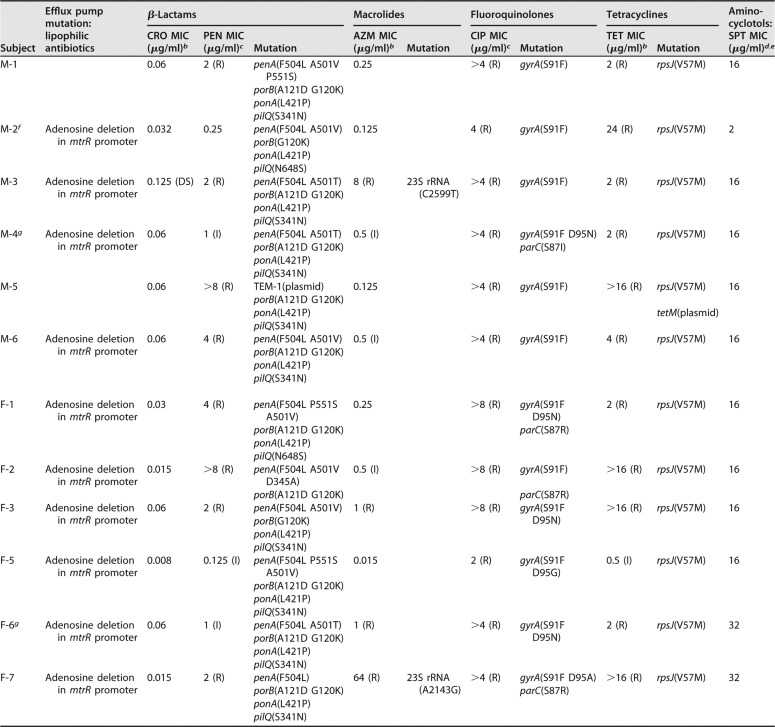
Phenotypic resistance and AMR genes identified in male and female N. gonorrhoeae isolates (*n* = 13) by WGS^a^

Subject	Efflux pump mutation: lipophilic antibiotics	β-Lactams			Macrolides		Fluoroquinolones		Tetracyclines		Amino- cyclotols: SPT MIC (μg/ml)^d,e^
		CRO MIC (μg/ml)^b^	PEN MIC (μg/ml)^c^	Mutation	AZM MIC (μg/ml)^b^	Mutation	CIP MIC (μg/ml)^c^	Mutation	TET MIC (μg/ml)^b^	Mutation	
M-1		0.06	2 (R)	*penA*(F504L A501V P551S)	0.25		>4 (R)	*gyrA*(S91F)	2 (R)	*rpsJ*(V57M)	16
				*porB*(A121D G120K)							
				*ponA*(L421P)							
				*pilQ*(S341N)							
M-2^f^	Adenosine deletion in *mtrR* promoter	0.032	0.25	*penA*(F504L A501V)	0.125		4 (R)	*gyrA*(S91F)	24 (R)	*rpsJ*(V57M)	2
				*porB*(G120K)							
				*ponA*(L421P)							
				*pilQ*(N648S)							
M-3	Adenosine deletion in *mtrR* promoter	0.125 (DS)	2 (R)	*penA*(F504L A501T)	8 (R)	23S rRNA (C2599T)	>4 (R)	*gyrA*(S91F)	2 (R)	*rpsJ*(V57M)	16
				*porB*(A121D G120K)							
				*ponA*(L421P)							
				*pilQ*(S341N)							
M-4^g^	Adenosine deletion in *mtrR* promoter	0.06	1 (I)	*penA*(F504L A501T)	0.5 (I)		>4 (R)	*gyrA*(S91F D95N)	2 (R)	*rpsJ*(V57M)	16
				*porB*(A121D G120K)				*parC*(S87I)			
				*ponA*(L421P)							
				*pilQ*(S341N)							
M-5		0.06	>8 (R)	TEM-1(plasmid)	0.125		>4 (R)	*gyrA*(S91F)	>16 (R)	*rpsJ*(V57M)	16
				*porB*(A121D G120K)							
				*ponA*(L421P)						*tetM*(plasmid)	
				*pilQ*(S341N)							
M-6	Adenosine deletion in *mtrR* promoter	0.06	4 (R)	*penA*(F504L A501V)	0.5 (I)		>4 (R)	*gyrA*(S91F)	4 (R)	*rpsJ*(V57M)	16
				*porB*(A121D G120K)							
				*ponA*(L421P)							
				*pilQ*(S341N)							

F-1	Adenosine deletion in *mtrR* promoter	0.03	4 (R)	*penA*(F504L P551S A501V)	0.25		>8 (R)	*gyrA*(S91F D95N)	2 (R)	*rpsJ*(V57M)	16
				*porB*(A121D G120K)				*parC*(S87R)			
				*ponA*(L421P)							
				*pilQ*(N648S)							
F-2	Adenosine deletion in *mtrR* promoter	0.015	>8 (R)	*penA*(F504L A501V D345A)	0.5 (I)		>8 (R)	*gyrA*(S91F)	>16 (R)	*rpsJ*(V57M)	16
				*porB*(A121D G120K)				*parC*(S87R)			
F-3	Adenosine deletion in *mtrR* promoter	0.06	2 (R)	*penA*(F504L A501V)	1 (R)		>8 (R)	*gyrA*(S91F D95N)	>16 (R)	*rpsJ*(V57M)	16
				*porB*(G120K)							
				*ponA*(L421P)							
				*pilQ*(S341N)							
F-5	Adenosine deletion in *mtrR* promoter	0.008	0.125 (I)	*penA*(F504L P551S A501V)	0.015		2 (R)	*gyrA*(S91F D95G)	0.5 (I)	*rpsJ*(V57M)	16
				*porB*(A121D G120K)							
				*ponA*(L421P)							
				*pilQ*(S341N)							
F-6^g^	Adenosine deletion in *mtrR* promoter	0.06	1 (I)	*penA*(F504L A501T)	1 (R)		>4 (R)	*gyrA*(S91F D95N)	2 (R)	*rpsJ*(V57M)	32
				*porB*(A121D G120K)							
				*ponA*(L421P)							
				*pilQ*(S341N)							
F-7	Adenosine deletion in *mtrR* promoter	0.015	2 (R)	*penA*(F504L)	64 (R)	23S rRNA (A2143G)	>4 (R)	*gyrA*(S91F D95A)	>16 (R)	*rpsJ*(V57M)	32
				*porB*(A121D G120K)				*parC*(S87R)			
				*ponA*(L421P)							
				*pilQ*(S341N)							

a CRO, ceftriaxone; PEN, penicillin; AZM, azithromycin; CIP, ciprofloxacin; TET, tetracycline; SPT, spectinomycin; S, susceptible; DS, decreased susceptibility; I, intermediate resistance; R, resistant.

b S = ≤0.25 µg/ml.

c S = ≤0.06 µg/ml.

d S = ≤32 µg/ml.

e No mutation was found to confer resistance to aminocyclotols.

f Isolate did not grow in agar dilution media, and MICs were determined by Etest on chocolate agar.

g Male and female partners in the dyad (M-4 and F-6).

Whole-genome DNA sequencing (WGS) was performed on all available strains (6 male and 6 female) to define strain relatedness and genotypic determinants of antibiotic resistance. A single-nucleotide polymorphism (SNP) distance tree was constructed that used SNP distances (differences) that ranged between 81 and 3,913 separating the genomes (see Fig. S2 in the supplemental material). The male and female dyad (M-4 and F-6) yielded genomic sequences that were separated by only 81 SNPs. Strain NCCP11945 isolated from a subject with gonococcal disease in South Korea (average SNP distance of 2,828 SNPs compared to strain FA1090) shared considerable homology with the isolates obtained from our cohort compared to the commonly used laboratory strain, FA1090 (average distance of 4,212 compared to FA1090).

10.1128/mSphereDirect.00312-18.2 FIG S2  Hamming distance tree and SNP distance matrix of N. gonorrhoeae isolates. Tree visualizing Hamming distances of total pairwise SNPs identified across strains. The scale bar shows a distance of 300 SNPs. Download FIG S2, TIF file, 0.9 MB. Copyright © 2018 Nudel et al. 2018 Nudel et al. This content is distributed under the terms of the Creative Commons Attribution 4.0 International license.

Genomic analyses based on publicly available, curated, antibiotic resistance databases (CARD and GC-MLST) identified multiple AMR genes (Table 2) and SNP-level variants that likely contributed to phenotypic resistance. All gonococcal strains exhibited SNP mutations in *gyrA* and/or *parC* (which confer resistance to fluoroquinolones), SNP mutations in *penA*, *ponA*, *porB*, and *pilQ* (β-lactam resistance), and SNP mutations in *rpsJ* (tetracycline resistance) (54). Only one isolate (M-5) carried plasmid-based resistance genes (*tem-1* and *tetM*). Interestingly, among the male subjects, M-3 was the only isolate to exhibit azithromycin resistance and a corresponding mutation in the 23S rRNA gene (55). Three isolates had an adenosine deletion in the promoter region of *mtrR*, the repressor of the *mtrCDE* efflux pump, a system that exports bactericidal host-derived compounds and hydrophobic antibiotics (β-lactams and macrolides) from the bacterial cell (56–58). Isolates from women exhibited similar genomic patterns of resistance: by and large, the same mutations identified in the male isolates were also present in the female isolates (Table 2). Among 6 SNPs associated with β-lactam resistance in the dyad isolates (subjects M-4 and F-6, who were infected by the same strain), 5 SNPs were shared in the dyad isolates. Two SNPs in *gyrA* were shared (S91F and D95N), and a Ser SNP in *parC* [*parC*(S87I)] was detected only in the male isolate. Finally, while several SNP mutations coincided with phenotypic resistance to azithromycin, ciprofloxacin, and penicillin (Table 2), the single isolate with decreased sensitivity to ceftriaxone (M-3) did not possess the mosaic *penA* XXXIV allele (59) or any additional curated mutations that distinguished it from the phenotypically sensitive strains (Table 2) (59, 60).

**FIG 4:**
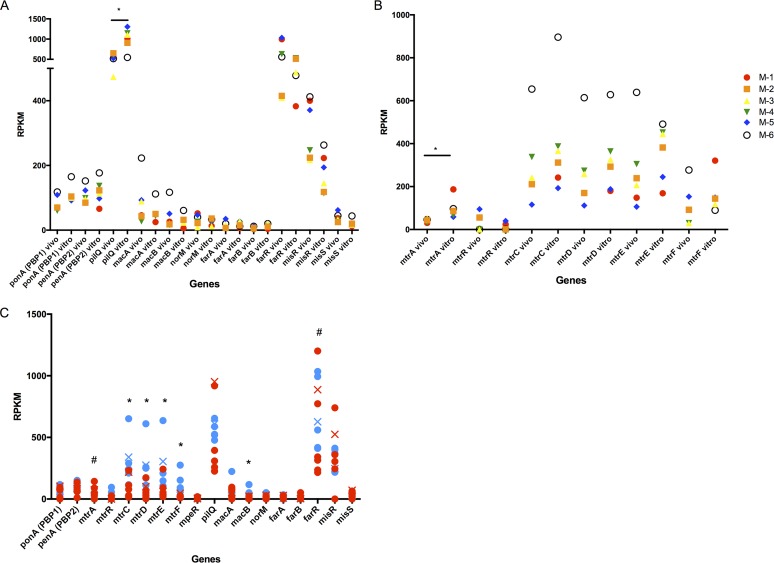
Expression of gonococcal antibiotic resistance genes. Expression levels of antibiotic resistance genes (A) and the Mtr efflux pump (B) in the male genital tract (*in vivo*) and the corresponding strain in CDM (*in vitro*); genes are identified on the *x* axis. (Six male specimens [*in vivo*] and the corresponding 6 isolates [*in vitro*] are shown in the same color.) *, *q* ≤ 0.05. (C) Expression levels of antibiotic resistance genes from the male genital tract (blue) or the female genital tract (red). *, higher gene expression in men; #, higher gene expression in women (*q* ≤ 0.05). Red and blue X’s indicate the male (M-4) and female (F-6) counterparts of the dyad.

Expression levels of genes associated with antibiotic resistance in men were also compared to expression of corresponding genes *in vitro* (Fig. 4A and B). Expression of the *pilQ* gene, associated with antibiotic permeability (14), was significantly decreased by 1.78-fold *in vivo* compared to *in vitro* (Fig. 4A). The MisRS two-component regulatory system is important for resistance to host-derived antimicrobial peptides and antibiotics (61), and though not statistically significant, *misR* had 2.08-fold higher expression *in vivo* compared to *in vitro*, supporting its important role *in vivo*. The expression of the Mtr efflux pump was also closely examined; expression of *mtrA*, which can influence expression of *mtrCDE* (62), was significantly decreased by 2.75-fold *in vivo* (Fig. 4B). Gonococcal strains from male urethral specimens (M-2, M-3, M-4, and M-6) contained an adenosine deletion in the *mtrR* promoter that was accompanied by the lowest expression of *mtrR* and highest expression of *mtrCDE* and *mtrF*, confirming that the adenosine deletion directly affects expression of the Mtr locus (63) *in vivo*. Expression of *mpeR* was also investigated, as it has been shown to regulate the Mtr locus; however, expression was below 10 RPKM in all *in vitro* and *in vivo* samples.

Transcriptomes from male urethral specimens were also compared with female cervicovaginal lavage specimens to determine if expression of AMR genes was influenced by sex (Fig. 4C). Because of the adenosine deletion in the *mtrR* promoter in 4/6 males (described above) and all female isolates (Table 2), expression of *mtrR* was low in both groups and resulted in significantly higher expression of *mtrCDE* and *mtrF* in men, as predicted (63), but not in women. Even within the dyad, where both M-4 and F-6 contained the adenosine deletion in the *mtrR* promoter, M-4 had approximately 2× higher expression levels of *mtrCDE* than F-6. Interestingly, the expression of *mtrA*, an activator of *mtrCDE* (62), was significantly higher during infection in women compared to men, despite lower expression of *mtrCDE* in women (Fig. 4C). The *farR* gene, which negatively controls expression of the *farAB* operon by directly binding to the *farAB* promoter (64) to prevent excess expression of gonococcal efflux pumps, also showed significantly higher expression in cervicovaginal lavages. Collectively these results suggest that, although strains isolated from infected men and women exhibit comparable AMR genotypes and *in vitro* phenotypes, expression of AMR genes is different during infection in the male and female genital tracts.

## DISCUSSION

N. gonorrhoeae infects the male and female genital tracts, two very distinct environments in humans. As there is little doubt about the intrinsic tissue, cellular, and molecular differences that define these host environments, it is reasonable to assume that the gonococcus will adapt to these environmental differences during infection (65). Building on our previous results on the gonococcal transcriptome profile during infection in women (37), we extended our analysis to specimens from infected men. In the present study, we not only report a distinct gonococcal transcriptome profile *in vivo* compared to *in vitro* in infected men, but also, for the first time, describe a comparison of N. gonorrhoeae gene expression during infection in the male and female genital tracts. Our results also demonstrate distinct gonococcal gene expression signatures in men and women, consistent with the intrinsically different makeups and natures of the two sites of infection. Furthermore, our approach based on both whole-genome and RNA sequencing enabled us to evaluate expression of antibiotic resistance determinants during infection in both the male and female genital tracts.

Currently, the only human model of gonococcal infection is experimental urethral challenge of male volunteers. Those studies have examined gonococcal virulence factors and have provided valuable information on opacity proteins and genes involved in iron acquisition, structural modification of lipooligosaccharides, and a variety of other established virulence factors (32, 66, 67) but are limited by the short duration of experimental infection. The small number of infecting strains used experimentally in men (mostly laboratory-adapted strains FA1090 and MS11mkC [32]) have precluded an in-depth understanding of the range of adaptions employed during gonococcal infections generally. Using a panel of N. gonorrhoeae strains isolated from infected subjects, we compared the gonococcal *in vivo* transcriptome expressed during infection of the male genital tract to that of the corresponding infecting strains grown *in vitro*. Our results revealed increased expression of genes involved in oxidative stress and iron scavenging *in vivo*, accompanied by decreased expression of genes involved in metabolism-associated categories (i.e., energy and amino acid metabolism). In particular, we observed that gonococci expressed high levels of *trx1*, *msrAB*, and glutaredoxin genes during infection in male subjects, in agreement with the large PMN influx that occurs during infection in symptomatic men. Our results also demonstrated increased expression of iron-regulated genes (*tbpAB* [66]), indicating that the male genital tract is an iron-depleted environment. We observed lower expression of genes involved in gonococcal aerobic energy metabolism processes and higher expression of oxidative stress response genes in urethral specimens compared to that of the corresponding infecting strains grown *in vitro*. It is established that the genital tract is anaerobic or microaerobic (68, 69). Thus, we would expect that a comparison of RNA-seq data sets from urethral and cervicovaginal lavage specimens to RNA-seq data sets from *in vitro* growth conditions would identify differential regulation of gonococcal genes utilized for aerobic energy production. However, the presence of neutrophils, especially in male subjects during infection, likely requires a corresponding N. gonorrhoeae oxidative defense response. Such immune cells are obviously absent under our *in vitro* conditions, and thus these oxidative stress pathways are expressed at lower levels. While we recognize that *in vitro* culture conditions differ compared to the conditions in the human genital tract beyond metabolite content, this study provides a gene-specific view of the response of N. gonorrheoae to infection and leads to a number of hypotheses regarding infection mechanisms that can be explored in more detail in future studies.

While we identified both expected and novel differences in gonococcal gene expression during human infection compared to *in vitro* growth, the most revealing aspect of our study was the differential gonococcal expression profiles observed in men and women. Our results showed that nearly 14% of gonococcal genes that were expressed in human infection were differentially expressed in men and women. Principal-component analysis demonstrated that gonococcal expression in men and women separated into distinct gene expression signatures, with high variance among the 7 female specimens and low variance among the 6 male specimens. We acknowledge that high variance could be due to the small sample size, but it may also be due to other female-specific environmental factors that could alter gonococcal gene expression, such as menstrual cycle or the microbiome, which exhibits diversity among individuals (70). We also observed differences in the presence and type of coinfecting organisms in infected men and women. Several male subjects had a coinfection of Chlamydia trachomatis or Mycobacterium genitalium, and while some female subjects were also infected with these pathogens, several females additionally carried Ureaplasma urealyticum. In addition, 3/7 of the female subjects had bacterial vaginosis (BV), suggesting differences in their natural microflora in combination with these coinfections. We hypothesize that changes in the microflora and the presence of coinfecting organisms have a direct effect on the transcriptomic response of N. gonorrhoeae, as does the variable host response during coinfection compared to infection with N. gonorrhoeae alone. Both of these factors most likely impact the overall differences observed between samples of the same gender as well as differences observed in the gonococcal transcriptomic response during infection in men and women. When genomic DNA sequence analysis was also considered, there was no clustering among strains derived from men or women (except for strains in the single dyad), which strongly suggests that the observed differences in transcriptomic signatures were driven by exposure of gonococci, generally, to the male or the female genital tract and not by strain relatedness. Furthermore, this is also supported in the single dyad by differences in gene expression manifested by the same strain in each partner, which reflected changes in gene expression that were seen overall in the other men and women. In addition to the small sample size, it is important to also consider other limitations of the study when interpreting sex-specific and growth-specific aspects of N. gonorrhoeae infection. Due to the design of the study, men are recruited because they enter the clinic with a symptomatic response: thus, men may have a similar stage of infection and gonococcal growth stage. This is in contrast to the matched females, who are partners of the recruited men and who were at various stages of infection and therefore gonococcal growth.

Among the most relevant gonococcal transcriptome differences that emerged in male and female specimens were those in genes encoding antimicrobial resistance determinants. The rise of antibiotic resistance in N. gonorrhoeae is now a global concern (71). Patterns of resistance vary among countries because of population mobility and geographically localized increases in antimicrobial resistance—such as those observed in China (72); these patterns are predictive of future resistance patterns in countries that currently have lower levels of resistance, such as the United States (73, 74). Whole-genome DNA sequencing (WGS) of the gonococcal strains isolated from our male and female cohort revealed similarity of genetic determinants of resistance to strains isolated in the United States (59). In all strains, regardless of the origin of the specimens (men or women), the presence of an adenosine deletion in the *mtrR* promoter (also detected by WGS) correlated with increased expression of the Mtr regulon. Nevertheless, strains isolated from men showed a significantly higher expression of *mtrCDE* and *mtrF* than those from women. While a direct explanation for these results remains speculative, it is possible that the higher expression of *mtrCDE* observed in men may be due to the readily accessible antibiotics in China; approximately half of the male subjects (but none of the female subjects) in this study had self-administered antibiotics prior to seeking care at the STD clinic. Future studies including a larger cohort may help resolve these differences. An additional explanation for gender-specific differences is that biofilm formation in the female genital tract (and apparent lack of it in the male genital tract) is a contributing factor (19, 20). Biofilms can provide protection against harsh environments, and their absence in the male genital tract may lead to increased expression of antibiotic-resistant efflux pumps as a defense mechanism (75, 76).

N. gonorrhoeae contains phage DNA inserted in the genome: five regions comprising double-stranded DNA (dsDNA) lysogenic phage genomes (50) and four additional regions comprising filamentous phages (77). The role of phage genes in N. gonorrhoeae infection has not been studied extensively. Previous work from our group has identified a phage gene, *npr*, involved in invasion of female epithelial cells *in vitro*, which correlates with disease progression in a female mouse model of gonococcal infection (78). Our finding that expression of phage genes was increased in female subjects may also relate to the formation of biofilms in the female genital tract (20). We propose that biofilm formation may facilitate DNA exchange via phages among bacteria, thus contributing to differences in infections of men and women. Additional transcriptional patterns that differentiated organisms obtained during infection in men and women included genes encoding tRNAs with higher expression observed in women compared to men. However, apparent expression of tRNAs may be partially affected by reads aligning to additional microbes present in the genital tract: tRNA genes are highly conserved across species, and the women in the study may have a larger population of microbial organisms. Six of seven gonococcus-infected women reported in this study had BV and/or other bacterial genital infections. Some studies have suggested a role for tRNAs in bacterial infection (79, 80), and it is possible that differences observed in men and women also correlate with the enhanced microbial environment in women. Small RNA expression *in vivo* was also different; NrrF and FnrS (an sRNA induced under anaerobic conditions) were expressed at higher levels in women than in men.

Collectively, our results provide the first global view of gonococcal gene expression during infection in humans and define gene signatures specific to infections in men and women. We report important differences related to antibiotic resistance and gonococcal pathogenesis that can be extrapolated to improve understanding of gonococcal disease outcomes and potential treatments. Our analysis also highlights shortfalls of studying bacterial infections using *in vitro* models and systems. It is critical that studies designed to identify targeted therapies for gonococcal infections consider sex-specific differences in gene expression profiles that may impact treatment outcomes. Furthermore, addressing how expression of antimicrobial resistance genes are driven by environmental cues in the male and female genital tracts has important implications for the use of targeted antibiotics.

## MATERIALS AND METHODS

### Ethics statement.

All subjects provided written informed consent in accordance with requirements by Institutional Review Boards from Tufts University, Boston, MA (protocol no. 11710), the University of Massachusetts Medical School, Worcester, MA, and Boston University School of Medicine, Boston, MA, and the Institute of Dermatology, Chinese Academy of Medical Sciences and Peking Union Medical College, Nanjing, China.

### Identification of subjects and characterization of urethral infections.

Urethral swab specimens were collected from six men attending the Nanjing Sexually Transmitted Disease (STD) Clinic, chosen at random, who were participants in a study of gonococcal transmission (81). Male subjects presented with symptoms and signs of urethritis (dysuria and/or urethral discharge). Gram stains of urethral exudates showed PMNs and Gram-negative intracellular diplococci, criteria that are highly specific (99%) for gonococcal infection (82). Urethral swab specimens from men with urethritis were also inoculated onto Thayer-Martin medium (DL Biotech, China) and cultured in candle jars at 36°C. Gonococcal isolates were identified by colonial morphology, Gram stain, and oxidase testing. Urethral swab specimens were also tested for Chlamydia trachomatis and Mycoplasma genitalium by PCR (83, 84) and Ureaplasma urealyticum and Mycoplasma hominis by culture using the *Mycoplasma* IST2 test (BioMérieux, France).

Collection of cervicovaginal lavage specimens (*n =* 4) was described previously (38), and three additional specimens were obtained for the present study (total *n =* 7). PCR testing using primers directed against the gonococcal *porA* pseudogene (85) had previously identified N. gonorrhoeae infection in one woman (F-4) whose cervical culture was negative. Women reported that they were monogamous (3/7 were married), having been the sole sex partner of a man with gonococcal urethritis. One woman (F-6) and one man (M-4) were members of a single dyad.

### Antimicrobial testing of N. gonorrhoeae*.*

N. gonorrhoeae strains from urethral swab and cervicovaginal lavage specimens were identified and processed as previously described (38). Antimicrobial sensitivity testing of N. gonorrhoeae isolates was determined for penicillin, tetracycline, spectinomycin, azithromycin, ceftriaxone, cefixime, and ciprofloxacin at the Nanjing STD Clinic. Mean inhibitory concentrations (MICs) were determined by the agar dilution method, and current Clinical and Laboratory Standards Institute cutoffs were used, except for azithromycin, in which we followed the European Committee on Antimicrobial Susceptibility Testing (http://www.eucast.org) (86). A single isolate (from M-2) that did not grow in agar dilution media or GC agar had MICs determined on chocolate agar by Etest: thus, MIC values may be one dilution lower due to enriched agar (87).

### RNA isolation.

Total RNA was isolated from urethral swab and cervicovaginal lavage specimens using TRIzol (Invitrogen) as described previously (38). Briefly, specimens were washed twice with 70% ethanol and treated with Turbo DNase (Ambion), the RiboMinus kit (Invitrogen) (to deplete eukaryotic rRNA), and the Microbe Express kit (Ambion) (to deplete bacterial rRNA) using diethylpyrocarbonate (DEPC)-treated EDTA-free water. Gonococcal isolates that corresponded to each specimen were grown overnight on chocolate agar plates at 37°C in 5% CO_2_ prior to inoculation in chemically defined medium (CDM) containing ferric nitrate (100 µM). Liquid cultures were inoculated at an optical density at 600 nm (OD_600_) of 0.1, harvested after 3 h, and pelleted, and RNA was extracted as described above.

### RNA sequencing and analysis.

RNA sequencing was performed as previously described (38). cDNA libraries were prepared with TruSeq RNA preparation kit and sequenced on an Illumina HiSeq 2500 instrument using high-output V3 chemistry in single-read 100 formats. Analysis of RNA-seq data was performed using the Rockhopper program, aligning the reads to the NCBI genome sequence of N. gonorrhoeae strain FA1090 and then reporting the RNA-seq data in reads per kilobase per million (RPKM) (37). The cutoff for gene expression was set at an RPKM value of at least 10. A composite set of RPKM values was derived from RNA-seq data from the six male urethral samples and another from the three newly collected cervicovaginal samples added to the four reported earlier (in the Gene Expression Omnibus database under GenBank accession no. GSE71151) (38). These are referred to as “*in vivo*” RNA-seq data. Data from gonococcal strains isolated from each subject and cultured *in vitro* were also obtained (referred to as “*in vitro*” data).

Statistical analysis was performed on pooled data from each category of specimens: *in vivo* urethral, *in vivo* cervicovaginal lavage, and *in vitro*. Genes with ≥2-fold change in expression and a *q* value of ≤0.05 in each category were deemed as regulated. Gene assignments and categorization were performed using the KEGG and NCBI RefSeq public databases. Functional enrichment ratios were determined by dividing the percentage of genes in a functional category for a particular set of genes (e.g., highly expressed genes or differentially regulated genes) by the percentage of genes in the same category present in the gonococcal genome as a whole. This ratio was used to interpret enrichment of a particular set of genes that possessed a specific function. Fisher’s exact test was used to assign a *P* value to the enrichment. Principal-component analysis (PCA) (88) was performed in R using singular value decomposition on the scaled and centered data set of RPKM values for all genes in each subject, and the resulting data were visualized by sex on a colored plot.

### Whole-genome sequencing and analysis.

N. gonorrhoeae isolates were grown on GC agar plates supplemented with 1% IsoVitaleX and 0.6% fetal bovine serum at 37°C and 5% CO_2_ for 16 to 18 h. Genomic DNA was extracted from a 10-µl inoculum of each bacterial culture using a 5Prime DNA extraction kit (5Prime, San Francisco, CA), following the manufacturer’s recommendations with slight modifications. Whole-genome sequencing (WGS) was conducted using a PacBio RSII platform (Pacific Biosciences, Menlo Park, CA) with P5-C3 chemistry. *De novo* genome assembly was conducted using the hierarchical genome assembly process workflow (HGAP3; SMRTAnalysis 2.3.0), which included consensus polishing using Quiver (89). Single-nucleotide polymorphisms (SNPs) across sequenced isolates were called *de novo*-assembled contigs using kSNP3 without a reference strain (90). Concatenated SNPs were used to construct phylogenetic trees using the approximate-maximum-likelihood-based approach; trees were visualized in FigTree (http://tree.bio.ed.ac.uk/software/figtree/). Antibiotic resistance elements were identified using the Comprehensive Antibiotic Resistance Database (91) and the PubMLST *Neisseria* database (https://pubmlst.org/neisseria) (92).

### Data availability.

New RNA-seq data from this study have been deposited in the Gene Expression Omnibus (GSE113290). Genomic data from isolates have been deposited into NCBI under project no. PRJNA329501 (see Table S2 in the supplemental material).

10.1128/mSphereDirect.00312-18.4 TABLE S2  Deposited genomic data. Download TABLE S2, DOCX file, 0.01 MB. Copyright © 2018 Nudel et al. 2018 Nudel et al. This content is distributed under the terms of the Creative Commons Attribution 4.0 International license.
