# Supplementary material for: Transcriptome Analysis of Neisseria gonorrhoeae during Natural Infection Reveals Differential Expression of Antibiotic Resistance Determinants between Men and Women
Source: mSphere. 2018 Jun 27;3(3):e00312-18. doi: 10.1128/mSphereDirect.00312-18 (PMC6021601; doi:10.1128/mSphereDirect.00312-18)
Supplement: TABLE S1 [file sph004182576st1.docx]

| **Table S1. Characteristics of Female Subjects** | | | |  |  |  |
| --- | --- | --- | --- | --- | --- | --- |
| Subject | Age | Days Since Male Contact | Days Since Menstrual Cycle | Other STI microbes | PMN Score** | Vaginal Wet Mount |
| F-1*** | 29 | 10 | 27 | None | ++ | Normal |
| F-2*** | 35 | 4 | 26 | *C. trachomatis, U. urealyticum, M. hominis* | ++ | Clue cells (BV+) |
| F-3*** | 21 | 11 | 16 | None | + | Clue cells (BV+) |
| F-4*** | 23 | 1 | 8 | *U. urealyticum* | None | Normal |
| F-5 | 24 | 8 | 27 | *C. trachomatis, U. urealyticum* | ++ | Normal |
| F-6 | 23 | 2 | 19 | *C. trachomatis, M. genitalium* | +++ | Normal |
| F-7 | 37 | 24 | 16 | None | + | Clue cells (BV+) |

| No female subject had had a prior *N. gonorrhoeae* infection or took antibiotics prior to the clinic visit |
| --- |
| **** Number of polymorphonuclear leukocytes (PMNs) / oil immersion field (oif) indicated: +, <5 PMNs; ++, 5-9 PMNs; +++, ≥10 PMNs |
| *** 4 female subjects have been described previously (McClure et al 2015 (38)); F-4 was diagnosed with gonorrhea by PCR. |
