# Supplementary figures and images for: Transcriptome Analysis of Neisseria gonorrhoeae during Natural Infection Reveals Differential Expression of Antibiotic Resistance Determinants between Men and Women
Source: mSphere. 2018 Jun 27;3(3):e00312-18. doi: 10.1128/mSphereDirect.00312-18 (PMC6021601; doi:10.1128/mSphereDirect.00312-18)

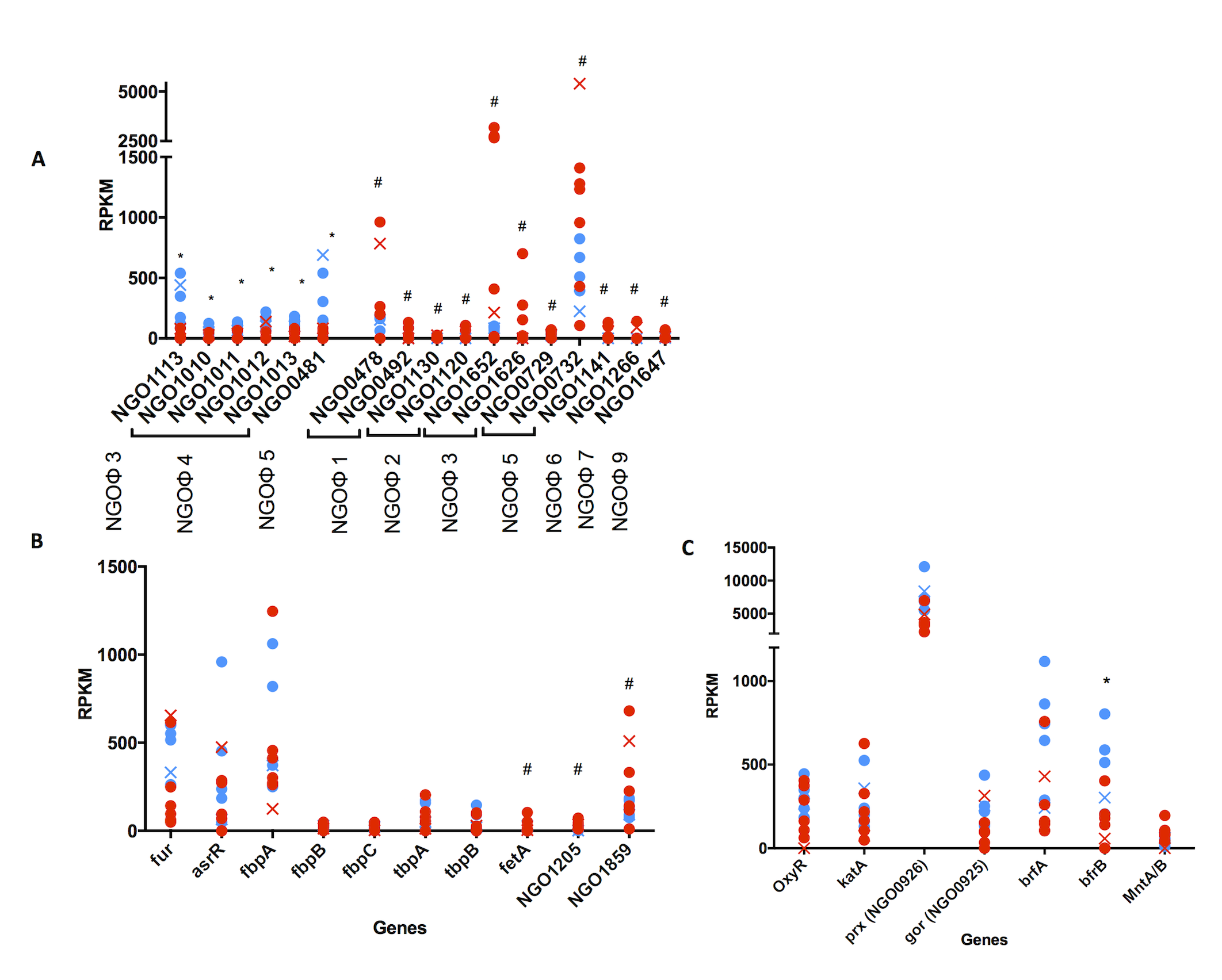

Supplement: FIG S1 [file sph004182576sf1.tif]

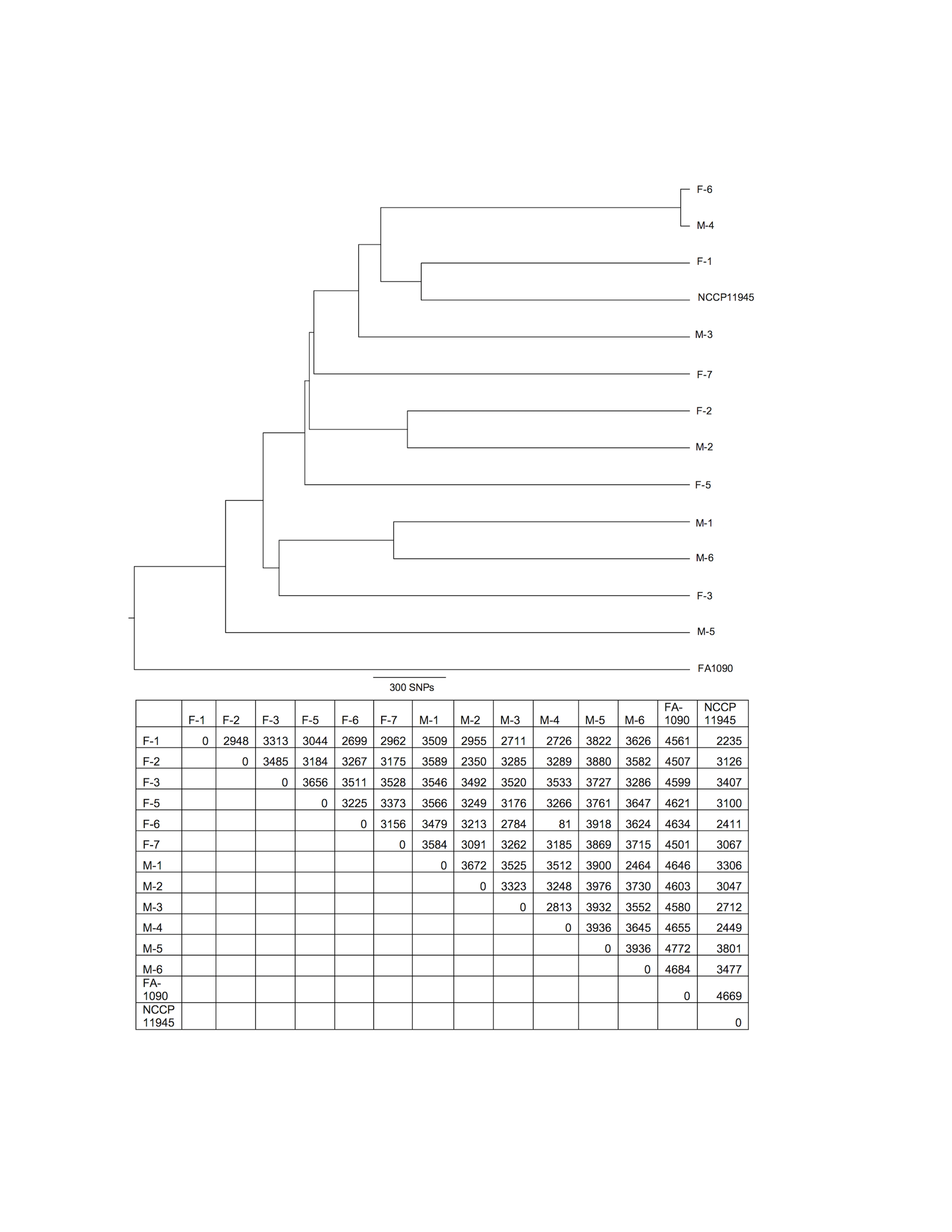

Supplement: FIG S2 [file sph004182576sf2.tif]
