# Supplementary material for: Transcriptome Analysis of Neisseria gonorrhoeae during Natural Infection Reveals Differential Expression of Antibiotic Resistance Determinants between Men and Women
Source: mSphere. 2018 Jun 27;3(3):e00312-18. doi: 10.1128/mSphereDirect.00312-18 (PMC6021601; doi:10.1128/mSphereDirect.00312-18)
Supplement: TABLE S2 [file sph004182576st2.docx]

| **Table S2. Deposited genomic data** | |  |  |  |
| --- | --- | --- | --- | --- |
| **Subject** | **Isolate Name** | **Mean Coverage (PacBio)** | **BioProject** | **BioSample #** |
| Female 1 | 05OP | 52 | PRJNA329501 | SAMN05413743 |
| Female 2 | 061Z | 148 | PRJNA329501 | SAMN05413744 |
| Female 3 | 04CD | 125 | PRJNA329501 | SAMN05413742 |
| Female 5 | GC_0706_75 | 63 | PRJNA329501 | SAMN05413759 |
| Female 6 | GC_07M2_73 | 120 | PRJNA329501 | SAMN05413757 |
| Female 7 | 03GN_87 | 162 | PRJNA329501 | SAMN05413739 |
| Male 1 | 0747_64 | 24 | PRJNA329501 | SAMN05413746 |
| Male 2 | 07AD_68 | 69 | PRJNA329501 | SAMN05413748 |
| Male 3 | 07D1_70 | 92 | PRJNA329501 | SAMN05413750 |
| Male 4 | GC_07MS_72 | 160 | PRJNA329501 | SAMN05413758 |
| Male 5 | GC_03A0_78 | 314 | PRJNA329501 | SAMN05413753 |
| Male 6 | GC-037F | 152 | PRJNA329501 | SAMN09059728 |
